# The effect of food insecurity on psychosocial aspects of academic achievement

**DOI:** 10.3389/fpubh.2026.1866556

**Published:** 2026-07-16

**Authors:** Meryem Elif Öztürk, Tuba Nur Yıldız Kopuz, Hasan Yıldırım

**Affiliations:** 1 Department of Nutrition and Dietetics, Faculty of Health Sciences, Karamanoglu Mehmetbey University, Karaman, Türkiye; 2 Department of applied mathematics, Kamil Özdag Science Faculty, Karamanoglu Mehmetbey University, Karaman, Türkiye

**Keywords:** academic achievement, food insecurity, psychosocial, stress, university student (MeSH)

## Abstract

**Introduction:**

Studies showed that food insecurity is associated with lower grade point average (GPA) in university students. However academic achievement should be investigated in psychosocial and multidimensional way. Therefore this study aimed to examine the stress mediated relationship between food insecurity and academic achievement, which is addressed from different perspectives.

**Methods:**

The study was conducted on 1,122 university students [seven cases were excluded as data-entry errors: four with implausible age values (2, 2, 167, 211 years) and three with implausible height values (16, 72, 104 cm)]. US Adult Food Security Survey Module, the Academic Success Inventory Scale for College Students, and the Perceived Stress Scale were applied to students.

**Results:**

Age was negatively associated with food insecurity [β = −0.143, OR = 0.87, 95% CI (0.79, 0.95), *p* = 0.002]. Compared with consuming no snacks, consuming one snack per day was associated with lower odds of food insecurity [β = −0.563, OR = 0.57, 95% CI (0.34, 0.96), *p* = 0.033]. Food insecurity was directly and negatively associated with perceived instructor efficacy [β = −0.091; 95% CI (−0.148, −0.034); *p* = 0.002, personal adjustment [β = −0.093; 95% CI (−0.145, −0.043); *p* < 0.001, and socializing [β = −0.160; 95% CI (−0.220, −0.103); *p* < 0.001] sub-dimensions. It was also associated with stress-mediated decreases in general academic skills (indirect β = −0.031; 95% CI [−0.048, −0.016], internal motivation (indirect [β = −0.029; 95% CI (−0.047, −0.015)], career decidedness (indirect [β = −0.025; 95% CI (−0.039, −0.012)], lack of anxiety (indirect β = −0.036; 95% CI (−0.054, −0.019)] and concentration (indirect β = −0.041; 95% CI (−0.062, −0.021)] sub-dimensions (all indirect-effect *p* < 0.001).

**Discussion:**

These findings emphasize that interventions to improve student achievement should consider access to food, stress management, and psychosocial support components.

## Introduction

In the Food and Agriculture Organization's (FAO) 2001 report, food security is defined as “a situation in which people lack secure access to sufficient, safe, and nutritious food necessary for normal growth and development, as well as for an active and healthy life (1).” This condition may result from the unavailability of food, insufficient purchasing power, or improper distribution or inadequate utilization of food at the household level. According to The Global Report on Food Crises 2025, in 2024, over 295 million people across 53 countries and territories suffered from acute hunger—an increase of approximately 14 million compared to 2023 (2). The report states that hunger and food insecurity problems have been increasing over the years around the world. Similarly, food insecurity in Türkiye has increased more than 1.5 times in the last 10 years (3). It was also found that higher levels of education, better health status and increased income are strongly associated with a lower risk of food insecurity, in Türkiye (3).

Food insecurity is prevalent in university students, households with children and people with health conditions (4, 5). In Türkiye food insecurity among college students varies between 33% and 68.2% which shows it is very common (6, 7). Among university students food insecurity was found to be heavily driven by sociodemographic factors such as low parental education, first-generation status, residence type, and lack of adequate financial aid (8). Also it was reported that food insecurity is related to skipping main meals especially breakfast and dinner, lower odds of snacking, healthy eating habits and healthy physical activity habits on campus (9, 10).

Food insecurity is associated with greater odds of having stress, depression and anxiety among university students (11, 12). It is also related to lower grade point average (GPA) scores (7). Food insecurity was found to be associated with lower academic performance with the mediating effect of mental stress (13). In studies examining the relationship between food insecurity and academic performance among university students, GPA scores are commonly used as indicators of academic achievement (7, 14). However, it has been recommended that abilities beyond GPA should also be measured to evaluate students' academic success more comprehensively (15).

In addition to cognitive skills—defined as the intellectual abilities that enable individuals to acquire, memorize, recall, compare, combine and use information–non-cognitive skills also play a significant role in academic success (16). These include motivation, self-regulation, self-discipline, self-efficacy, confidence, tenacity, self-appraisal, social skills, and communication skills (17).

A better understanding of academic success at the university level requires the development of complex models that incorporate various social, cognitive, and interpersonal variables, as well as interdisciplinary theoretical concepts (18). Therefore, we used the Academic Success Inventory for College Students (ASICS) to comprehensively assess academic success. This allowed us to measure a wide range of psychometric dimensions related to academic success, including general academic skills, perceived instructor efficacy, internal motivation/confidence, external motivation/future orientation, personal adjustment, socializing, career decidedness, lack of anxiety, and concentration. The aim of the study is to examine the stress-mediated relationship between food insecurity and academic achievement by addressing academic success from these multiple dimensions.

## Materials and methods

This cross sectional study was conducted on university students from various cities of Türkiye [predominantly in the middle and south of the country) with snowball sampling method between May and June 2025. Initially 1,135 students participated into the study. Six students were excluded due to missing data. And seven cases were excluded as data-entry errors: four with implausible age values (2, 2, 167, 211 years) and three with implausible height values (16, 72, 104 cm)]. Therefore the final analytic sample size was 1,122. Including criteria were; being a university student and able to speak Turkish. After the research *post-hoc* power analysis was conducted. The power of the study (1–β) was found to be 92% (Alpha 5% and confidence interval 95%). This study complied with the principles of the Declaration of Helsinki. The study was approved by the Ethics Committee of Karamanoglu Mehmetbey University (ethics approval code 05-2025/71). Students were administered a questionnaire that included questions on sociodemographics, eating habits, and lifestyle, the US Adult Food Security Survey Module, the Academic Success Inventory Scale for College Students, and the Perceived Stress Scale. Anthropometric measurements were obtained from students. Body Mass Index (BMI) was calculated.

### Sociodemographics, eating habits and lifestyle

We questioned sociodemographic characteristics, eating habits, and lifestyle factors, including gender, age, maternal education, paternal education, maternal working status, paternal working status, residence type, dieting (Yes/No), vitamin and mineral supplement intake (Yes/No), daily main meal frequency, daily snack frequency, skipping main meals (which main meal is most frequently skipped: breakfast, lunch, or dinner), and regular physical activity (at least three times a week and at least 30 min at once) (Yes/No). Daily meal frequency and snack frequency were initially asked as an open-ended question, then converted into a categorical variable. Snack frequency was modeled as a 4-level categorical variable (none [reference]; one; two; three or more snacks per day), with the original 0–6 count collapsed at ≥3 because cells beyond three were sparse (*n* = 4, 3, 1 for 4, 5, and 6 snacks/day respectively). Income was estimated based on participants' responses: income equal to expenses, income less than expenses, and income more than expenses. These variables were included because previous literature suggests that sociodemographic characteristics and lifestyle-related factors may influence both food insecurity and academic outcomes and may therefore act as potential confounding factors. We assessed demographic characteristics to better understand the parental and sociocultural backgrounds of the students. Although meal skipping is included in the food security survey as an indicator, we assessed it separately to evaluate the potential relationship between meal frequency, food insecurity and academic success.

### US adult food security survey module

The ten–item US Adult Food Security Survey Module (AFSSM) was developed by US Department of Agriculture (USDA). It assesses the food security status of an individual adult rather than a household with children (19). In the questionnaire, five items are yes/no questions, while the other five items use the response options: often true, sometimes true, and never true. Turkish validity and reliability was established by Açar et al. (20). The Turkish version comprises 8 items and uses an additive scoring scheme yielding a total score ranging from 0 to 48; item-level scoring follows the protocol described by Açar et al. (20). A score of 0–10 indicates food security, while a score of 11–48 indicates food insecurity.

### Academic success inventory scale for college students

The scale was developed by Prevatt et al. (18) to evaluate academic success in college students. It includes 50 items and 10 sub-dimensions, with responses collected using a 7-point Likert scale ranging from 1 (strongly disagree) to 7 (strongly agree). The Turkish validity and reliability of the scale were established by Orçanli et al. (21). The Turkish version comprises 46 items and 9 sub-dimensions: general academic skills, perceived instructor efficacy, internal motivation/confidence, external motivation/future, personal adjustment, socializing, career decidedness, lack of anxiety, and concentration.

Higher scores indicate better academic performance. For instance, higher general academic skills scores reflect stronger study skills, effort, and self-regulation strategies. Internal motivation/confidence is associated with a stronger belief in one's ability to succeed academically, while external motivation/future reflects awareness of the course's relevance for future goals. High personal adjustment scores suggest the absence of personal issues that interfere with academic performance. Similarly, high socializing scores indicate a balanced level of social activity or alcohol consumption that does not negatively affect academic success. Career decidedness refers to progress toward a well-defined career objective.

### Perceived stress scale

The 14-item scale was developed by Cohen et al. (22). It was designed to measure the degree to which individuals perceive certain situations in their lives as stressful. The Turkish validity and reliability of the study was established by Eskin et al. (23). Participants rate each item on a 5-point Likert scale ranging from “Never (0)” to “Very Often (4).” Reverse scoring of the seven positively-worded items (items 4, 5, 6, 7, 9, 10, 13) was applied prior to summation, such that in the analytic dataset all items are coded in the direct (stress) direction with higher scores indicating higher perceived stress. The total score, computed on the original 0–4 response convention, ranges from 0 to 56; in the present sample the observed total ranged from 8 to 54 (Cronbach's α = 0.74).

### Statistical analysis

The statistical evaluation of the data was performed using R software (version 4.4.1) and MedCalc Statistical Software (version 23.0.9). For all analyses, statistical significance was accepted at a *p*-value of less than 0.05. Initially, the data were summarized using descriptive statistics. Continuous variables were presented as mean ± standard deviation (SD), while categorical data were reported as frequencies (*n*) and percentages (%).

A binomial logistic regression model was constructed to identify the factors predicting food insecurity. The model's parameters were estimated using the Maximum Likelihood Estimation (MLE) method. Several diagnostic tests were performed to ensure the model's assumptions were met. The assumption of linearity between the continuous predictors and the logit of the outcome variable was checked using the Box-Tidwell test. Multicollinearity among independent variables was assessed by calculating the Variance Inflation Factor (VIF) and Tolerance statistics, ensuring VIF < 10 and Tolerance > 0.1. Influential outliers were identified by examining Cook's distance (with > 1.0 as the threshold for concern) and leverage values; diagnostic checks indicated no influential cases requiring removal (Cook's distance: max = 0.010; leverage: max = 0.73). Snack frequency was modeled as a 4-level categorical variable (none [reference], one, two, three or more snacks/day); the original count (range 0–6) was collapsed at ≥3 due to sparse cell counts beyond three.

The direct association between food insecurity and academic performance sub-dimensions was analyzed through a series of simple linear regression models, with both unstandardized (b) and standardized (β) coefficients reported alongside their 95% confidence intervals. The assumptions for linear regression were rigorously checked. Residual plots (residuals vs. fitted values) were visually inspected to confirm linearity and homoscedasticity (constant variance of errors), with the latter being formally tested using the Breusch-Pagan test. The independence of residuals was evaluated with the Durbin-Watson test, and the normality of residuals was verified using Q-Q plots and the Shapiro-Wilk test.

Finally, mediation analyses were conducted to determine if stress mediated the association between food insecurity and the academic-achievement sub-dimensions. For each outcome we estimated, in standardized form, (i) the total effect of food insecurity, (ii) the direct effect controlling for stress, (iii) the indirect effect through stress, and (iv) the proportion mediated. The significance of all path coefficients was tested using a non-parametric bootstrapping procedure with 5,000 resamples (percentile method), generating 95% confidence intervals (CI); an effect was deemed statistically significant when its CI did not include zero. Proportion mediated was reported only when the total and indirect effects shared the same direction; for outcomes with opposite-signed direct and indirect components (inconsistent mediation), proportion mediated is not interpretable and is denoted as such. This approach is robust to the non-normality of indirect-effect sampling distributions.

## Results

In the present study, 73.1% of students with valid food-security data (*n* = 815) were food secure and 26.9% (*n* = 300) were food insecure (7 cases had missing food-security data). The demographic characteristics of the 1,122 students in the analytic sample show that 68.89% were female (*n* = 773) and 31.11% were male (*n* = 349). The average age of the participants was 21.76±1.95. Regarding parental education levels, 52.50% of mothers were literate or had completed primary education (*n* = 589), and 38.59% had completed secondary education (*n* = 433). Data on the participants' housing status indicate that 68.45% were living in dormitories (Table 1).

**Table 1:** The summary characteristics of participants (*n* = 1,122).

Main characteristics		N(%)
Gender	Male	349 (31.11%)
	Female	773 (68.89%)
Age; mean±SD		21.77 ± 1.95
BMI; mean±SD		22.86 ± 3.57
Maternal education; *n* (%)	Literate and primary education	589 (52.50%)
	Secondary education	433 (38.59%)
	Bachelor's degree and above	97 (8.65%)
Paternal education; *n* (%)	Literate and primary education	408 (36.36%)
	Secondary education	517 (46.08%)
	Bachelor's degree and above	191 (17.02%)
Maternal working status	Housewife	766 (68.27%)
	Employed	278 (24.78%)
	Retired	75 (6.68%)
Paternal working status	Not employed	50 (4.46%)
	Employed	708 (63.10%)
	Retired	358 (31.91%)
Grade	Preparatory class	39 (3.48%)
	First grade	226 (20.14%)
	Second grade	297 (26.47%)
	Third grade	230 (20.50%)
	Fourth grade	330 (29.41%)
Residence type	Dorm	768 (68.45%)
	Other off–campus housing	204 (18.18%)
	Parent's home	150 (13.37%)
Income status	Income equal to expenses	615 (54.81%)
	Income less than expenses	254 (22.64%)
	Income more than expenses	251 (22.37%)
Dieting	Yes	183 (16.31%)
	No	935 (83.33%)
Vitamin and mineral supplements usage	Yes	321 (28.61%)
	No	798 (71.12%)
Daily Main meal frequency	One main meal	39 (3.48%)
	Two main meals	735 (65.51%)
	Three main meals	348 (31.02%)
Daily Snack frequency	None	136 (12.12%)
	One	418 (37.25%)
	Two	419 (37.34%)
	Three and more	149 (13.28%)
Skipping main meals	Breakfast	295 (34.42%)
	Lunch	527 (61.49%)
	Dinner	35 (4.08%)
Regular physical activity	Yes	512 (45.63%)
	No	607 (54.10%)

A logistic regression analysis was conducted to identify the demographic, socioeconomic, and lifestyle factors predicting food insecurity among university students. The assumption of multicollinearity in the model was assessed using Variance Inflation Factor (VIF) and Tolerance values. For all predictor variables, the VIF values were below 1.17, and the Tolerance values were above 0.853. Since these values fall within acceptable limits (typically VIF < 10, Tolerance > 0.1), it was concluded that there is no multicollinearity problem in the model. The results of the tests conducted to evaluate the overall fit and significance of the logistic regression model indicate that the model fits the data significantly well (χ^2^ (25) = 99.23, *p* < 0.001).

Age has a negative and significant effect on food insecurity (β = −0.143, *p* = 0.002). Each additional year of age reduces the likelihood of experiencing food insecurity by 13.6% [Odds Ratio (OR) = 0.867, 95% Confidence Interval (CI) (0.790, 0.951)]. Compared to the reference group whose mothers had “literacy or primary education,” students whose mothers had completed “secondary education” were 40.3% less likely to experience food insecurity [β = −0.516, *p* = 0.014; OR = 0.597, 95% CI (0.396, 0.900)]. Students whose fathers were retired retained significantly lower odds of food insecurity compared with those whose fathers were unemployed [β = −0.828, OR = 0.437, 95% CI (0.216, 0.884), *p* = 0.021], with the effect estimate slightly stronger than in the original analysis. Compared to students who reported their economic status as “income less than expenses” (reference group), those who reported their economic status as “income equal to expenses” [β = −0.972, *p* < 0.001; OR = 0.378, 95% CI (0.258, 0.554)] and “income greater than expenses” [β = −1.206, *p* < 0.001; OR = 0.299, 95% CI (0.176, 0.510)] were respectively 62.2% and 70.1% less likely to experience food insecurity. Compared to students who did not consume any snacks (“none” as the reference group), those who consumed “one snack” per day (β = −0.563, *p* = 0.033; OR = 0.569), and “two snacks” per day (β = −0.986, *p* < 0.001; OR = 0.373) were significantly less likely to experience food insecurity. This result indicates that food insecurity is related to less snack frequency among students. Variables including BMI, gender, paternal education level, maternal employment status, type of residence, dieting, use of vitamin-mineral supplements, number of main meals, and skipping main meals were not statistically significantly associated with food insecurity (*p* > 0.05); however, regular physical activity emerged as a borderline-significant correlate after outlier filtering [β = −0.394, OR = 0.675, 95% CI (0.477, 0.954), *p* = 0.026, with non-physically-active students serving as the reference], a counter-intuitive direction. The analytic sample for this model was *n* = 841 (281 cases were excluded by listwise deletion, primarily on the conditional ‘skipping main meals' item which is only completed by those who skip meals); diagnostic checks based on Cook's distance (max = 0.011) and leverage (max = 0.13) indicated no influential cases warranting removal (Table 2).

**Table 2:** Predictors of food insecurity among university students: results from a binomial logistic regression.

Main characteristics	β	SE	Z	*p*	Odds ratio (OR)	95% CI of Odds	
						Lower	Upper
**Intercept**	3.962	1.283	3.090	0.002	52.559	4.252	649.730
**Age**	−0.143	0.047	−3.030	0.002	0.867	0.790	0.951
**BMI**	0.003	0.007	0.470	0.640	1.003	0.989	1.018
Gender							
Male–Female	0.166	0.201	0.820	0.410	1.181	0.796	1.751
Maternal education							
Secondary education–(Illiterate-primary)	−0.516	0.210	−2.460	0.014	0.597	0.396	0.900
Bachelor's degree and above–(Illiterate-primary)	−0.054	0.379	−0.140	0.888	0.948	0.451	1.993
Paternal education							
Secondary education–(Illiterate-primary)	0.021	0.205	0.100	0.918	1.021	0.683	1.527
Bachelor's degree and above–(Illiterate-primary)	−0.242	0.296	−0.820	0.415	0.785	0.439	1.404
Maternal working status							
Retired—Housewife	0.439	0.348	1.260	0.207	1.551	0.785	3.065
Employed–Housewife	0.250	0.212	1.180	0.237	1.285	0.849	1.945
Paternal working status							
Retired—Not employed	−0.828	0.360	−2.300	0.021	0.437	0.216	0.884
Employed—Not employed	−0.613	0.347	−1.770	0.077	0.542	0.274	1.070
Residence type							
Dorm–Parent's home	0.315	0.281	1.120	0.261	1.371	0.791	2.375
Other off-campus housing—Parent's home	0.092	0.338	0.270	0.785	1.097	0.565	2.129
Income status							
Income equal to expenses—Income less than expenses	−0.972	0.195	−4.990	< 0.001	0.378	0.258	0.554
Income more than expenses—Income less than expenses	−1.206	0.272	−4.440	< 0.001	0.299	0.176	0.510
Dieting							
Yes–No	−0.075	0.228	−0.330	0.742	0.928	0.594	1.450
Vitamin/Mineral supplement intake							
Yes-No	−0.196	0.194	−1.010	0.314	0.822	0.562	1.203
Daily main meal frequency							
Two main meals–one main meal	0.103	0.400	0.260	0.797	1.108	0.506	2.426
Three main meals–one main meal	−0.110	0.475	−0.230	0.817	0.896	0.353	2.273
Daily snack frequency							
One—None	−0.563	0.264	−2.140	0.033	0.569	0.340	0.955
Two—None	−0.986	0.276	−3.570	< 0.001	0.373	0.217	0.641
Three—None	−0.643	0.341	−1.880	0.061	0.526	0.269	1.026
Skipping main meals							
Lunch—Breakfast	−0.037	0.190	−0.200	0.844	0.963	0.664	1.398
Dinner—Breakfast	0.587	0.418	1.410	0.159	1.799	0.794	4.079
Regular Physical activity							
Yes–No	0.397	0.176	2.250	0.025	1.487	1.048	2.097
**Overall model test**	χ^2^				*p*		
	99.23				< 0.001		

P < 0.05 indicates statistical significance.

The fundamental and direct relationship between food insecurity and various components of academic performance were examined through regression analysis. As food insecurity increased, students' “General Academic Skills” significantly decreased (b = −0.113, standardized β = −0.061, *p* = 0.043). The negative association with “Perceived Instructor Efficacy” (b = −0.066, standardized β = −0.105, *p* < 0.001) indicates that students experiencing food insecurity perceive the educational support provided by their teachers as less effective. At the motivational level, both “Internal Motivation” (b = −0.033, standardized β = −0.071, *p* = 0.017) and “External Motivation” (b = −0.033, standardized β = −0.061, *p* = 0.050) were negatively associated with food insecurity. Food insecurity was related to lower personal adjustment (b = −0.065, standardized β = −0.142, *p* < 0.001), indicating that food insecure students'personal problems affect their academic performance adversely (Table 3).

**Table 3:** Associations between food insecurity and academic achievement subdimensions in university students.

Dependent	Dependent sub level	Predictor	Unstandardized b and standardized β (95% CI), *p*
**Academic achievement**	**General academic skills**	**Food insecurity**	**b** ** = −0.113 (unstandardized);** **β** ** = −0.061 (standardized, 95% CI:−0.119,−0.002);** ***p*** **=** **0.043**
	**Perceived instructor efficacy**		**b** ** = −0.066 (unstandardized);** **β** ** = −0.105 (standardized, 95% CI:−0.164,−0.047);** ***p*** **<** **0.001**
	**Internal Motivation/Confidence**		**b** ** = −0.033 (unstandardized);** **β** ** = −0.071 (standardized, 95% CI:−0.130,−0.013);** ***p*** **=** **0.017**
	**External Motivation/Future**		**b** ** = −0.032 (unstandardized);** **β** ** = −0.059 (standardized, 95% CI:−0.117**, **+0.000);** ***p*** **=** **0.050**
	**Personal adjustment**		**b** ** = −0.065 (unstandardized);** **β** ** = −0.142 (standardized, 95% CI:−0.200,−0.083);** ***p*** **<** **0.001**
	**Socializing**		**b** ** = −0.072 (unstandardized);** **β** ** = −0.166 (standardized, 95% CI:−0.224,−0.108);** ***p*** **<** **0.001**
	**Career decidedness**		**b** **=** **+0.000 (unstandardized);** **β** **=** **+0.000 (standardized, 95% CI:−0.059**, **+0.059);** ***p*** **=** **0.994**
	**Lack of anxiety**		**b** **=** **+0.014 (unstandardized);** **β** **=** **+0.030 (standardized, 95% CI:−0.028**, **+0.089);** ***p*** **=** **0.311**
	**Concentration**		**b** ** = −0.005 (unstandardized);** **β** ** = −0.015 (standardized, 95% CI:−0.074**, **+0.044);** ***p*** **=** **0.612**

**Figure 1:**
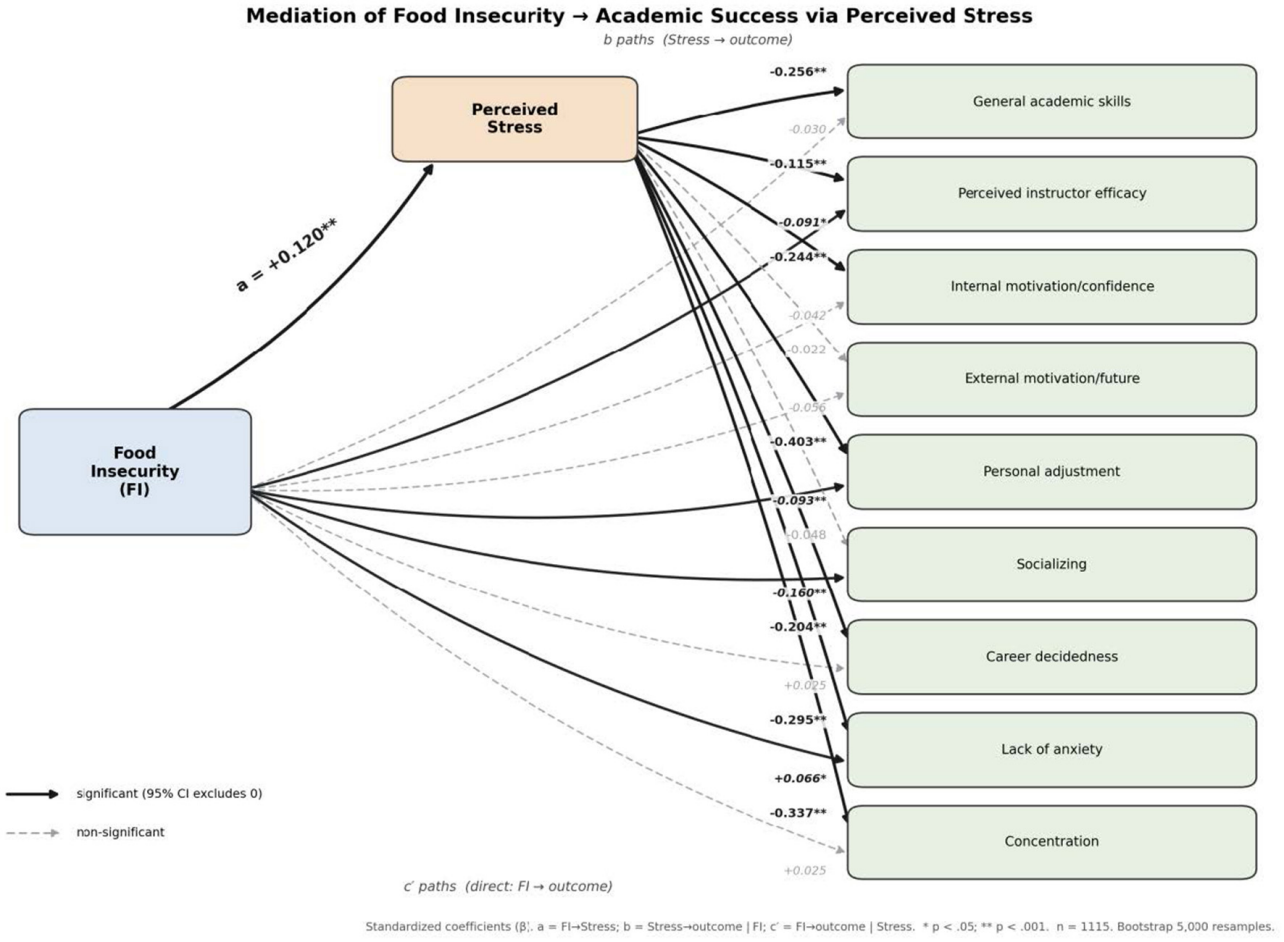
Mediation path diagram of food insecurity on the nine ASICS sub-dimensions of academic success through perceived stress. All coefficients are standardized (β): a = food insecurity → stress; b = stress → outcome (adjusted for food insecurity); c′ = direct food insecurity → outcome path (adjusted for stress). Solid bold arrows mark significant paths (95% CI excludes 0); dashed gray arrows mark non-significant paths. * *p* < 0.05; ** *p* < 0.001. The a path is common to all outcomes (β = 0.12). *n* = 1,115; 5,000 bootstrap resamples. * =*p*<0.05; ** =*p*<0.001.

**Figure 2:**
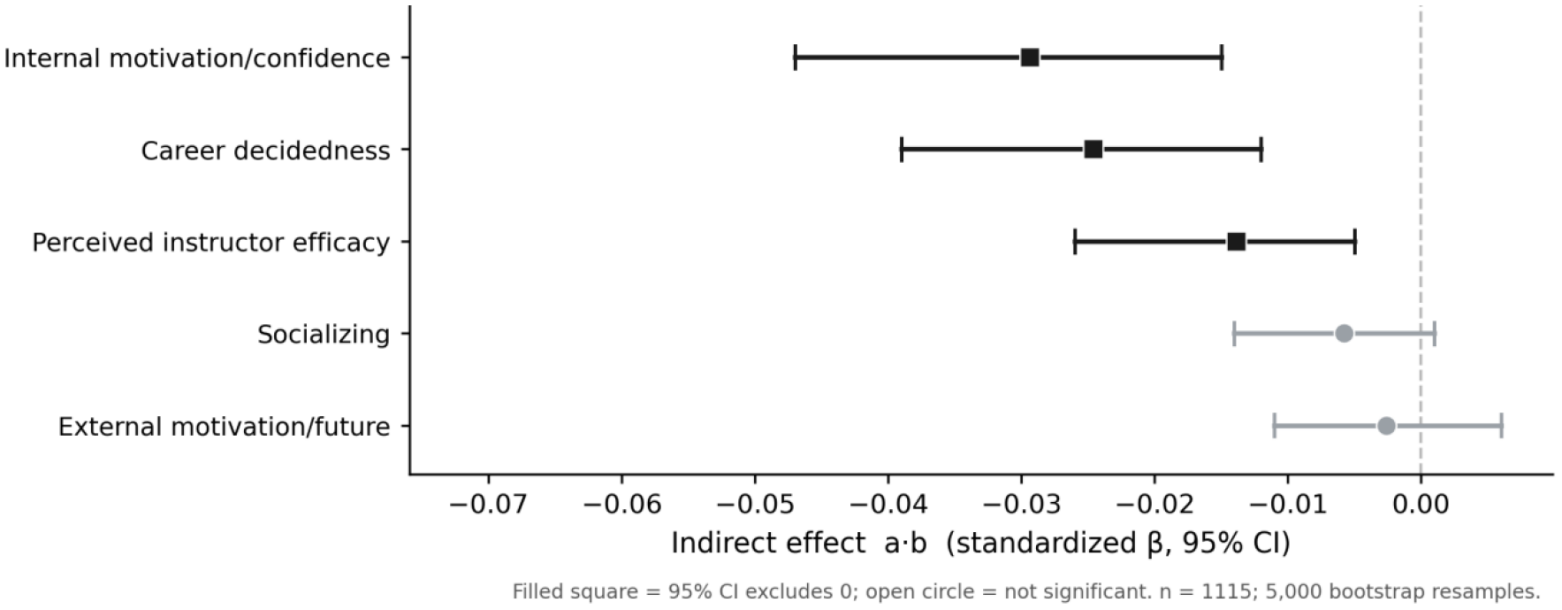
Indirect effect (a•b) of food insecurity on each ASICS sub-dimension through perceived stress, in standardized β with 95% CI. Filled squares denote outcomes whose CI excludes 0 (significant); open circles denote non-significant outcomes. 5,000 bootstrap resamples.

According to the SEM, food insecurity was associated with higher stress scores [β = 0.120; 95% CI (0.067, 0.176); *p* < 0.001], and stress was associated with lower general academic skills [β= −0.256; 95% CI (−0.316, −0.194); *p* < 0.001]. The direct effect of food insecurity on general academic skills was not significant [β= −0.030; 95% CI (−0.090, 0.030); *p* = 0.308], but the indirect effect, mediated by stress, was significant [β= −0.031; 95% CI (−0.048, −0.016); *p* < 0.001]. Both the direct and indirect effects of food insecurity on perceived instructor efficacy were significant [β= −0.091; 95% CI (−0.148, −0.034); *p* = 0.002, and β = −0.014; 95% CI (−0.026, −0.005); *p* < 0.001, respectively]. We also found that food insecurity had a significant indirect effect on internal motivation, mediated by stress [β = −0.029; 95% CI (−0.047, −0.015); *p* < 0.001]. However, after accounting for stress, food insecurity had neither a direct [β = −0.056; 95% CI (−0.119, 0.006); *p* = 0.063] nor an indirect effect [β = −0.003; 95% CI (−0.011, 0.006); *p*=0.548] on external motivation. Both the direct and indirect effects of food insecurity on personal adjustment were significant [β= −0.093; 95% CI (−0.145, −0.043); *p* < 0.001, and β= −0.048; 95% CI (−0.072, −0.027); *p* < 0.001]. Food insecurity had a significant direct effect on socializing [β= −0.160; 95% CI (−0.220, −0.103); *p* < 0.001], but no significant indirect effect mediated by stress [β= −0.006; 95% CI (−0.014, 0.001); *p*=0.106]. Lastly, the indirect effects of food insecurity on career decidedness [β= −0.025; 95% CI (−0.039, −0.012); *p* < 0.001], lack of anxiety [β= −0.036; 95% CI (−0.054, −0.019); *p* < 0.001], and concentration [β = −0.041; 95% CI (-0.062,−0.021); *p* < 0.001], all mediated by stress, were significant (Figures 1, 2). Notably, for three outcomes — career decidedness, lack of anxiety, and concentration — the total effect of food insecurity on the outcome was not statistically significant despite a significant negative stress-mediated indirect effect; this pattern reflects inconsistent mediation, in which the direct and indirect components are of opposing signs (or the direct path is essentially null while the indirect path is non-zero), and the proportion mediated is therefore not interpretable. Table 4 presents the full decomposition (total, direct, indirect, and proportion mediated) for all nine sub-dimensions.

**Table 4:** Mediation analysis of stress in the association between food insecurity and academic-achievement sub-dimensions (standardized coefficients with bootstrap 95% CI, 5,000 resamples).

ASICS sub-dimension	a path: FI → stress (95% CI)	b path: stress → outcome (95% CI)	Total effect c (95% CI)	Direct effect c' (95% CI)	Indirect effect ab (95% CI)	Prop. mediated	Mediation type
General academic skills	+0.120 (0.067, 0.176)^**^	**-**0.256 (-0.316,−0.194)^**^	−0.061 (-0.119,−0.002)^*^	−0.030 (-0.090, 0.030) n.s.	−0.031 (-0.048,−0.016)^**^	0.51	Full mediation
Perceived instructor efficacy	+0.120 (0.067, 0.176)^**^	−0.115 (-0.178,−0.051)^**^	−0.105 (-0.164,−0.047)^**^	−0.091 (-0.148,−0.034)^*^	−0.014 (-0.026,−0.005)^**^	0.13	Partial mediation
Internal motivation/confidence	+0.120 (0.067, 0.176)^**^	−0.244 (-0.304,−0.181)^**^	−0.071 (-0.130,−0.013)^*^	−0.042 (-0.098, 0.017) n.s.	−0.029 (-0.047,−0.015)^**^	0.41	Full mediation
External motivation/future	+0.120 (0.067, 0.176)^**^	−0.022 (-0.086, 0.044) n.s.	−0.059 (-0.117, 0.000) n.s.	−0.056 (-0.119, 0.006) n.s.	−0.003 (-0.011, 0.006) n.s.	—	No mediation
Personal adjustment	+0.120 (0.067, 0.176)^**^	−0.403 (-0.456,−0.344)^**^	−0.142 (-0.200,−0.083)^**^	−0.093 (-0.145,−0.043)^**^	−0.048 (-0.072,−0.027)^**^	0.34	Partial mediation
Socializing	+0.120 (0.067, 0.176)^**^	−0.048 (-0.106, 0.011) n.s.	−0.166 (-0.224,−0.108)^**^	−0.160 (-0.220,−0.103)^**^	−0.006 (-0.014, 0.001) n.s.	0.04	Direct effect only
Career decidedness	+0.120 (0.067, 0.176)^**^	−0.204 (-0.270,−0.139)^**^	+0.000 (-0.059, 0.059) n.s.	+0.025 (-0.036, 0.082) n.s.	−0.025 (-0.039,−0.012)^**^	n/a	Inconsistent mediation^†^
Lack of anxiety	+0.120 (0.067, 0.176)^**^	−0.295 (-0.354,−0.237)^**^	+0.030 (-0.028, 0.089) n.s.	+0.066 (0.004, 0.125)^*^	−0.036 (-0.054,−0.019)^**^	n/a	Inconsistent mediation^†^
Concentration	+0.120 (0.067, 0.176)^**^	−0.337 (-0.397,−0.276)^**^	−0.015 (-0.074, 0.044) n.s.	+0.025 (-0.034, 0.083) n.s.	−0.041 (-0.062,−0.021)^**^	n/a	Inconsistent mediation^†^

^*^p < 0.05; ^**^ p < 0.001 (CI excludes zero). ^†^Inconsistent mediation: direct and indirect effects have opposite signs; proportion mediated is not interpretable. The path (food insecurity → perceived stress) is common to all models: β = 0.120, 95% CI [0.067, 0.176]. n.s., non significant.

To convey the magnitude of these associations on the original measurement scale, Table 5 reports the observed mean ± SD on each ASICS sub-dimension for food-secure and food-insecure students, together with the Welch-corrected mean difference (95% CI), the corresponding *p*-value, and Cohen's d as a standardized effect-size estimate. The largest practical effect was observed for socializing (d = 0.39, small-to-medium), followed by personal adjustment (d = 0.29) and perceived instructor efficacy (d = 0.26), both falling in the small range; the remaining six sub-dimensions showed effect sizes of trivial magnitude (|d| < 0.20), indicating that — despite reaching statistical significance under a large sample — several of the associations correspond to modest raw-scale differences in students' self-reported academic functioning.

**Table 5:** Observed ASICS sub-dimension scores by food-security status, with mean differences (Welch's *t*-test) and Cohen's d.

ASICS sub-dimension	Food-secure (M ±SD), *n* = 815	Food-insecure (M ±SD), *n* = 300	Mean difference (95% CI)	*p*	Cohen's d
General academic skills	75.54 ± 19.89	74.24 ± 19.91	+1.30 (-1.34, +3.94)	0.334	+0.07 (trivial)
Perceived instructor efficacy	21.04 ± 6.63	19.34 ± 6.72	+1.70 (+0.81, +2.58)	< 0.001	+0.26 (small)
Internal motivation/confidence	23.01 ± 4.87	22.49 ± 5.15	+0.52 (-0.15, +1.19)	0.131	+0.11 (trivial)
External motivation/future	21.03 ± 5.61	20.41 ± 6.03	+0.62 (-0.16, +1.41)	0.119	+0.11 (trivial)
Personal adjustment	11.82 ± 4.89	10.42 ± 4.71	+1.40 (+0.77, +2.03)	< 0.001	+0.29 (small)
Socializing	17.63 ± 4.35	15.86 ± 4.96	+1.77 (+1.13, +2.41)	< 0.001	+0.39 (small–medium)
Career decidedness	13.97 ± 4.84	14.24 ± 4.83	−0.27 (-0.91, +0.37)	0.412	−0.06 (trivial)
Lack of anxiety	9.56 ± 4.77	9.85 ± 5.16	−0.29 (-0.96, +0.38)	0.399	−0.06 (trivial)
Concentration	11.63 ± 3.37	11.60 ± 3.40	+0.03 (-0.42, +0.48)	0.881	+0.01 (trivial)

Cohen's d magnitude convention: |d| < 0.20 trivial, 0.20–0.49 small, 0.50–0.79 medium, ≥ 0.80 large. Group sizes correspond to the analytic sub-sample with complete stress and ASICS data (n = 1,115).

## Discussion

This study found that approximately one-quarter of university students experience food insecurity, food insecurity was associated with poorer outcomes across several dimensions of academic performance, and that stress plays a significant mediating role in this relationship. These findings suggest that food insecurity is not merely an economic problem, but is also associated with poorer academic achievement, with psychosocial factors potentially contributing to this relationship.

### Food insecurity among university students

This study found that 27.2% of university students experience food insecurity. This rate is similar to the 28.0% prevalence reported by the FAO at the global level (24). However, data on the prevalence of food insecurity in Türkiye are limited. Existing studies have reported moderate to severe food insecurity rates ranging from 26.5% to 47.6% among adults (25, 26). and from 33% to 68.2% among university students (6, 7). Similarly, studies from other countries have reported prevalence estimates ranging from 14% to 62.8% among university students (27, 28). This wide frequency range may reflect differences in study populations, geographic regions, and assessment methods. Niyaz reported a prevalence rate of 33% among university students in northwestern Türkiye (6), while Esin and Ayyildiz reported a prevalence rate of 68.2% in a nationwide sample collected via online snowball sampling (7). Although the present study was conducted at a university located in Central Anatolia, participants were recruited from different regions of the country through snowball sampling. Furthermore, food insecurity has been assessed using different instruments across studies, including the Household Food Security Questionnaire Module (HFSSM) and the Food Insecurity Experience Scale (FIES). This may further contribute to the differences in reported prevalence estimates.

Our findings indicate that food insecurity decreases as university students get older. Previous studies have shown that food insecurity risks are increasing, particularly among upper-class students and transfer students increases (29, 30). One possible explanation for this discrepancy is that the characteristics and living conditions of university students may differ across institutions and countries, which could influence their risk of experiencing food insecurity. Additionally, our study found a negative relationship between snack consumption and food insecurity. This finding is consistent with the results of another study conducted on university students in Türkiye (31). The literature also reports that students experiencing food insecurity tend to skip meals and consume energy-dense, nutrient-poor snacks more frequently (32, 33). However, studies reporting greater snack consumption among food-insecure individuals have generally focused on the nutritional quality of snacks, whereas the present study evaluated snack frequency. This difference in measurement may partly explain the apparent discrepancy between findings.

Contrary to expectations, students who engaged in regular physical activity were found to have higher food insecurity scores. This can be explained by the fact that physically active individuals perceive available food resources as insufficient due to their increased energy and nutrient requirements. Also, students may have to walk to school because they cannot use public transportation due to budget constraints (34). However, due to the cross-sectional design of the study, a causal interpretation is not possible.

### Food insecurity and academic achievement

This study found that food insecurity was associated with poorer outcomes across multiple dimensions of academic performance. Similarly, previous studies have reported that university students experiencing food insecurity demonstrate lower academic achievement. Weaver et al. and Tin et al. found a significant relationship between food insecurity and lower grade point averages (35, 36). Ahmad et al. reported in their study conducted in Malaysia that students experiencing food insecurity had lower cumulative grade point averages and reported higher levels of stress, anxiety, and depression (27). The underlying mechanisms of the relationship between food insecurity and academic achievement have not been clearly explained. However, it is thought that food insecurity is associated with poorer physical (37, 38), insufficient sleep (37, 39, 40), and poor mental health (37, 39, 41); food insecurity has been associated with poorer cognitive functioning and learning-related outcomes (27, 41, 42). Most studies in the literature examining the relationship between food insecurity and academic achievement have assessed academic performance solely through grade point average (27, 35–37, 43). Our findings suggest that food insecurity is associated not only with academic achievement but also with several psychosocial dimensions related to the learning process.

In addition, food insecurity was associated with lower socializing scores, one of the subdimensions of academic achievement. However, the socializing dimension of the ASICS reflects balanced social engagement, including appropriate levels of socializing and alcohol consumption that do not interfere with academic performance (21). Therefore, lower socializing scores should not be interpreted simply as reduced socialization, but rather as lower balanced socializing. Previous studies have reported that poor socialization negatively affects academic adjustment and increases the likelihood of dropping out of school (44, 45). Berger et al. demonstrated that the organizational component of socialization has a negative effect on dropping out of classes, while the relational component of socialization has a positive effect on academic performance (44). However, consistent with our findings, it has been reported that socializing, which increases negative habits such as alcohol consumption, negatively affects the academic performance of university students (46, 47). Therefore, the effect of socialization is not one-dimensional; positive socialization involving belonging and supportive relationships enhances academic achievement, while unbalanced socialization that increases negative habits such as excessive alcohol consumption can have the opposite effect.

### The mediating role of perceived stress

The study findings reveal that stress mediates the relationship between food insecurity and academic achievement. SEM analyses show that food insecurity was associated with higher perceived stress levels and that higher stress levels were associated with poorer performance in many dimensions, such as general academic skills, perceived instructor efficacy, motivation, personal adjustment, concentration, and career decidedness. This situation is consistent with evidence that food insecurity is associated with poorer mental health and cognitive functions as a “toxic stressor” (48). Previous studies suggest that unmet basic needs may contribute to chronic stress, which impairs cognitive processes such as energy levels, attention, decision-making, and learning, potentially contributing to poorer academic performance (48, 49). Raskind et al. demonstrated that the effect of food insecurity on academic performance primarily manifests through depression, anxiety, and low hope (14). Similarly, Brownfield et al. confirmed in Australia that psychological distress partially mediates this relationship (50). Our findings also highlight stress as a key component of this mechanism. Stress, anxiety, and depression associated with food insecurity may be linked to lower self-esteem and reduce their desire to attend classes (51). Psychological effects such as decreased self-esteem in students experiencing food insecurity may indirectly affect how they perceive the effectiveness of their instructors. Previous studies have reported an association between nutrition and academic motivation (52, 53). Our findings indicate that stress may play a role in the association between food insecurity and internal motivation/confidence. Although the indirect effect sizes observed in the present study were relatively modest, statistically significant associations were identified across multiple dimensions of academic achievement. Given that academic success is a multidimensional construct encompassing academic skills, motivation, concentration, social adjustment, and career-related factors (18), these findings may still be relevant when considered collectively across domains.

## Conclusion

Our study suggests that food insecurity is associated with poorer outcomes across multiple dimensions of academic performance among university students. Food insecurity was associated with poorer outcomes in general academic skills, internal motivation/confidence, personal adjustment, concentration, perceived instructor efficacy, and socializing. SEM further suggested that several of these associations were mediated by stress. These findings emphasize that interventions to improve student achievement should consider access to food, stress management, and psychosocial support components.

### Strengths and limitations

This study has several important strengths. First, academic performance was assessed using multidimensional ASICS rather than relying solely on a single indicator such as grade point average, thereby providing a more comprehensive evaluation of academic functioning. Furthermore, SEM was used to test the mediating role of stress; this approach contributes significantly to understanding the underlying psychosocial mechanisms of food insecurity's effects on academic performance.

However, there are also some limitations. Participants were selected using snowball sampling. While this method facilitated the inclusion of a large number of students, it may have introduced selection bias and reduced the sample's representativeness. The cross-sectional design of the study does not allow for causal inference about the relationship between food insecurity, stress, and academic performance. Data were collected using self-report scales, and participants' responses may be subject to recall bias and social desirability bias. Additionally, academic achievement was assessed using the ASICS, a self-report measure. Therefore, the findings reflect students' perceptions of their academic functioning and achievement, which may differ from objectively measured academic performance. Furthermore, stress is a multidimensional construct, and the Perceived Stress Scale assesses subjective perceptions of stress rather than physiological responses. Finally, the study was conducted at a single university, which may limit the generalizability of the findings to university students in other regions or educational settings.

## Data Availability

The raw data supporting the conclusions of this article will be made available by the authors, without undue reservation.
